# Probing the Bottleneck of Awareness Formed by Foveal Crowding: A Neurophysiological Study

**DOI:** 10.3390/brainsci14020169

**Published:** 2024-02-07

**Authors:** Ziv Siman-Tov, Maria Lev, Uri Polat

**Affiliations:** School of Optometry and Vision Sciences, Bar-Ilan University, Ramat Gan 5290002, Israel; zivst2@gmail.com (Z.S.-T.); maria.lev@biu.ac.il (M.L.)

**Keywords:** EEG, crowding, masking, grouping, tagging, pop-out, visual processing, awareness

## Abstract

Crowding occurs when an easily identified isolated stimulus is surrounded by stimuli with similar properties, making it very difficult to identify. Crowding is suggested as a mechanism that creates a bottleneck in object recognition and awareness. Recently, we showed that brief presentation times at the fovea resulted in a significant crowding effect on target identification, impaired the target’s color awareness, and resulted in a slower reaction time. However, when tagging the target with a red letter, the crowding effect is abolished. Crowding is widely considered a grouping; hence, it is pre-attentive. An event-related potential (ERP) study that investigated the spatial–temporal properties of crowding suggested the involvement of higher-level visual processing. Here, we investigated whether ERP’s components may be affected by crowding and tagging, and whether the temporal advantage of ERP can be utilized to gain further information about the crowding mechanism. The participants reported target identification using our standard foveal crowing paradigm. It is assumed that crowding occurs due to a suppressive effect; thus, it can be probed by changes in perceptual (N1, ~160 ms) and attentive (P3 ~300–400 ms) components. We found a suppression effect (less negative ERP magnitude) in N1 under foveal crowding, which was recovered under tagging conditions. ERP’s amplitude components (N1 and P3) and the behavioral proportion correct are highly correlated. These findings suggest that crowding is an early grouping mechanism that may be combined with later processing involving the segmentation mechanism.

## 1. Introduction

The phenomenon in which the ability to recognize an object is reduced when it is surrounded by similar objects, known as crowding, has been widely studied. Crowding has recently been interpreted as optimal integration given the limited precision of peripheral processing [1]. Crowding is considered a form of inhibitory interaction between the surrounding nearby contours and the target and produces “a fundamental limit on conscious perception and object recognition” [2]. Recent data demonstrate that visual crowding is correlated with awareness [3]. One of the crowding theories is called Gestalt grouping [4,5,6]. To perceive the whole object, the local features (grouping) of an object must be combined. Nador and Reeves [7] found that peripheral crowding is eliminated when both spatial frequency and the orientation of the Gabor flankers differ from those of the Gabor target, but changing just one of them has almost no effect. If the local and basic features comply with the Gestalt principles [8,9], they can be combined. However, previous studies have shown that in the periphery, crowding is reduced when the target and flankers are dissimilar in shape, size, orientation, polarity, spatial frequency, depth, color, and motion (tagging and pop-out effects) [10,11,12,13,14,15]. Similarly, the fovea of strabismic amblyopes, which is considered as periphery, typically has extensive crowding, but tagging the target in foveal crowding experiments revealed that crowding is largely reduced [2,16]. Thus, when some features differ from others, the grouping process is disrupted. This hypothesis can explain how crowding is reduced in the periphery and fovea by tagging. These results strengthen the hypothesis that Gestalt grouping [4,5,6] is one factor among others for crowding. The classic term ‘pop-out’ may not accurately describe the tagging effect, so we prefer to use tagging as a more appropriate term.

There are two main hypotheses regarding information processing strategies in crowding: one suggests that crowding is bottom-up and pre-attentive based on the concept that crowding occurs at a lower visual processing level [17]. Dakin et al. (2009) suggested that crowding is not influenced by attentional limit, and that crowding and attention do not share the same neural mechanisms [9]. Specifically, crowding occurs even when people are completely unaware of the flankers; this suggests that awareness and attention are not early conditions for crowding [10,18,19].

Another perspective suggests that crowding is a top-down process and that an attentional process is based on the idea that crowding occurs at a higher processing level. Therefore, the attentional resolution in peripheral vision limits the access of crowded targets to the consciousness [20,21]. Yeshurun and Carrasco showed that attention improves performance at peripheral locations by enhancing spatial resolution [22]. Furthermore, attention reduces the critical target–flanker distance at which the flankers no longer interfere with target identification [23]. The accumulated data lead to the conclusion that crowding occurs at multiple stages in the visual hierarchy [10].

The crowding phenomenon has been explored using human behavior (psychophysical, PSY) [2,24,25] and in event-related potential (ERP) by Chicherov and Herzog (2014) and others [26,27,28,29,30], mainly in peripheral crowding studies. Some studies revealed the foveal crowding effect using adaptive optics or under brief presentation times [31,32,33]. To date, there have been many psychophysical studies on crowding (for a review, see Levi 2007 [2]), but only a few have used ERP to study foveal crowded conditions [34]. These studies showed that the main effect of peripheral or foveal crowding is suppression, displayed by the N1 ERP component [28,34]. Therefore, the N1 amplitude increased as the crowding effect decreased [34]. In contrast, the earlier component, P1, is assumed to be correlated with a physical energy stimulus and is not affected by behavior; since it appears very early, it should not be affected by crowding [34]. The main conclusion of Chicherov and Herzog’s (2014) study is that crowding occurs during higher-level visual processing. Another interpretation is related to the assumption that the N1 amplitude represents facilitatory or inhibitory lateral interactions [35]; therefore, the source of N1 is considered as early or intermediate visual processing [35,36]. A recent EEG study measured the N1 amplitude under peripheral crowding as well as uncrowding under attended and unattended conditions [30].

Most of the studies on pop-out were performed in the periphery of the visual field, assuming that this phenomenon directs attention to an area of interest where the participant is not looking directly [37]. Many studies have shown a high correlation between the P3 component, attention, and awareness. Its amplitude was found to increase with increasing attention and awareness resources [38,39,40,41,42,43]. In addition, one of the characteristics of P3 is that it is affected by attention and decision making [44]. Another recent study suggested that crowding occurs simultaneously across multiple levels of visual processing [45]. In addition, it was suggested that crowding occurs automatically and that it can be modulated by attention.

In our previous study [31], using brief presentation times (40, 80, 120, and 240 ms), participants with normal monocular and binocular vision were asked to indicate the direction of the E target under isolated and crowded conditions. The stimulus was presented at the fixation point in the center of vision (fovea) to reveal the crowding effect. We found that crowding significantly reduces sensitivity under monocular and binocular conditions (*p* < 0.0001). The sensitivity under crowding conditions increases as the presentation time increases. We also used a red letter relative to the background (black matrix) to isolate the target [12,31]. There was a significant improvement in the sensitivity at all presentation times. 

We found that the foveal crowding effect can be reduced (to almost the level of the uncrowded condition) by tagging the target letter with a different color. Very interestingly, in further research [46], we showed that under a very brief presentation time, the participant’s perception of the target’s color (black or red) was impaired; in other words, in many trials, the participants were not aware about the target color, but crowding was still negated. Moreover, the reaction time under crowded conditions was markedly slower; however, the RT rebound that occurred even when the tag color was invisible showed that attentional and decision factors are not involved. It was suggested that the representation of an object consists of representations of many of the relevant aspects of it, and these are likely to be distributed in different cortical locations [47]. Thus, we wanted to explore the electrophysiological markers of early and late processing (attention and decision) using an ERP experiment that may shed light on the open question of whether there is a common mechanism for crowding and tagging (pop-out). Hence, our main hypothesis was that, since tagging decreases the foveal crowding effect, this effect would consequently increase the N1 amplitude, improve attention and decision making, and therefore also increase the P3 amplitude.

## 2. Methods

### 2.1. Participants

Ten adults with normal or corrected-to-normal vision and with no known neurological disorders participated in this study. Using the Ishihara test, we ensured that the participants did not have color deficiencies. The EEG data of one participant were removed from the analyses due to a technical failure during the experiment. The ages of the participants were between 20 and 38 (mean = 27.22, SD = 6.34). Visual functions were tested by a certified optometrist before participation in the study. All participants had a visual acuity of at least 20/20 and all were right-handed.

The participants signed a consent form that was approved by the Internal Review Board (IRB) of Bar-Ilan University, and all methods were performed according to the relevant guidelines and regulations; each participant was included only after ‘informed’ consent was obtained. All study protocols were approved by the Ethics Committee of Bar-Ilan University.

### 2.2. Stimuli and Procedures

**Figure 1:**
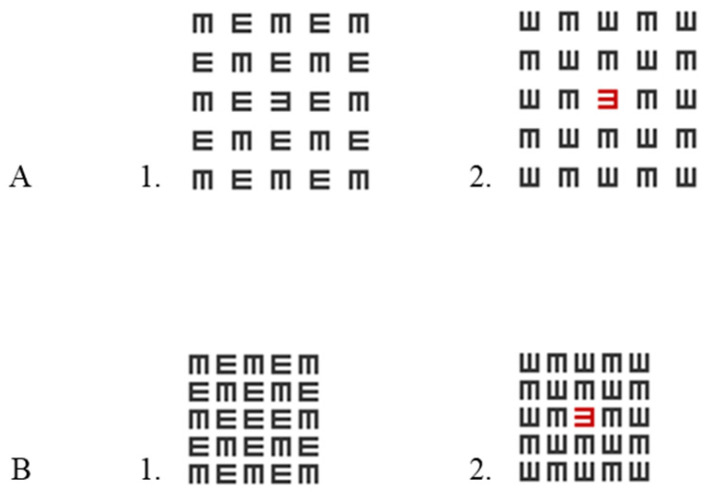
Stimuli. (**A**) 1. A black target letter in a black matrix with crowding of one-letter spacing (black uncrowded, BUC). 2. A red tagged letter in a black matrix with crowding of one-letter spacing (red uncrowded tagging, RUC). (**B**) 1. A black target letter in a black matrix with crowding of 0.4-letter spacing (black crowded, BC). 2. A red tagged letter in a black matrix with crowding of 0.4-letter spacing (red crowded tagging, RC). Note that the target letter is the letter in the center of the matrix.

The sitting distance was fixed at 185 cm. We measured the luminance intensity of the stimulus (E letter-isolated/crowding) and the background (a white screen) using a luminance meter (LS-100 KONICA MINOLTA, Tokyo, Japan). The luminance of both the black and red of the target letter and matrix was 9.5 cd/m^2^; it was presented on 65 cd/m^2^ of a white screen. The Weber contrasts were 85%. All stimuli were displayed binocularly. There was a matrix of E letters around the E target letter (black or red), which was arranged randomly. The size of the matrix was 5 × 5 letters. The size of each matrix letter corresponded to the size of the target letter. The target letter was an E letter presented at the center of the screen (presented on the central fovea), and it was marked by a fixation point. The letter E was displayed at a size of 2.7 mm, corresponding to a visual acuity of 20/20 (i.e., the stroke width was 1 arcminute, and the overall size of the letter was 5 arcminutes). The target letter was shown either in black or red. The task of the participants was to indicate the direction of the E target by clicking on the mouse key by using two different fingers (right click for E or left click for ꓱ); counterbalancing across participants was not performed because, in our opinion, this is the intuitive way. The side of the button corresponded to the side to which the signal was directed. In our further research, we did not find a significant difference between a black matrix with a red target letter and a red matrix with a black target letter, so we chose to present only the first condition [46]. There were four conditions (in random order [mixed by trials]): two for uncrowded (1-letter spacing between letters and a total size of 35 arcminutes) conditions (black, red) and two for crowded (0.4-letter spacing between letters and a total size of 23 arcminutes) conditions (black, red; in the middle of a black matrix) (see Figure 1). Note that the 0.4-letter spacing represents 0.4 times the letter size when the spacing is measured from edge to edge. Our ‘uncrowded’ definition relies on a previous article [48], and the spacing was carried out according to the methodology of visual acuity testing using the ETDRS chart where there is a space of one letter between letters to avoid crowding. Unlike in our previous article [31], here, we presented a spaced matrix (1-letter spacing) instead of a single letter to maintain a similar energy level (for ERP waves) for a smaller matrix (0.4-letter spacing).

For each condition, the stimulus was presented at one presentation time (30, 40, 50, 60, or 80 ms) that was adapted to each participant according to their detection threshold (60–80% for the black crowded condition). All experiments were administered in a dark room and were performed on the same day. Each run lasted about 4 min continuously without a break, but participants were allowed to take a break without any time limit between the runs, which were tested on 5 repetitions for each participant. There were 150 trials/conditions for each participant (i.e., 1500 trials/conditions for ten participants). We used a jitter of 0.4-letter spacing, i.e., all the stimuli were presented at the center of the screen with four possible identifications of letter rotations (up, down, right, and left) at random. The participants were instructed to respond quickly, unless the response speed led to errors. Although the reaction time data were not the main component of our study, they were used as references for the other results. The reaction times were measured for each trial from the moment the stimulus appeared until the subject clicked on the mouse key; trials in which no press was made were not included in the calculation of the reaction times.

### 2.3. Apparatus

Stimuli were presented on a 23.5″ (53.3 × 30 cm) LCD monitor (ASUS VG248QE) with a 100 Hz refresh rate and at a 1920 × 1080 pixel resolution using an NVIDIA GeForce GT 730 graphic card. The visual angle of the LCD monitor was 16.4° × 9.3°. The monitor had high temporal accuracy and was found to be suitable for visual psychophysics. The stimuli were presented using an in-house-developed platform for psychophysical experiments (PSY) developed by Yoram Bonneh and run on a Windows PC [49]. Note that the platform knows how to identify time missed and can filter those instances.

#### EEG Recording and Processing

**Figure 2:**
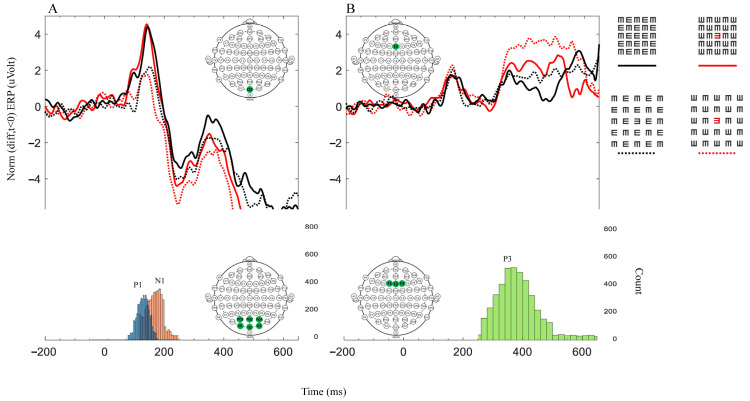
Each graph represents a different brain area denoted by a green electrode in the EEG inserted montage. Graph (**A**) represents the occipital electrodes (PO3, POZ, PO4, O1, OZ, and O2), and graph (**B**) represents the frontal electrodes (F1, FZ, and F2). The upper graphs represent the average (*n* = 9) ERP data for the correct response results. Each color and line type of the four lines represent a different matrix (on the upper right side of the graphs). The solid black line denotes BC, and the solid red line denotes RC. The dotted black line denotes BUC, and the dotted red line denotes RUC. The bottom graphs correspond to all conditions and represent the distribution of the count (the number of peaks) for the three components, namely P1 (blue), N1 (orange), and P3 (green), to ensure that the upper ERP graph faithfully represents the main components. The time windows are 50–200 ms for P1, 150–250 ms for N1, and 250–650 ms for P3. The y-axis represents the baseline corrected to the mean value of −200 ms, which precedes the stimulus onset data points.

**Figure 3:**
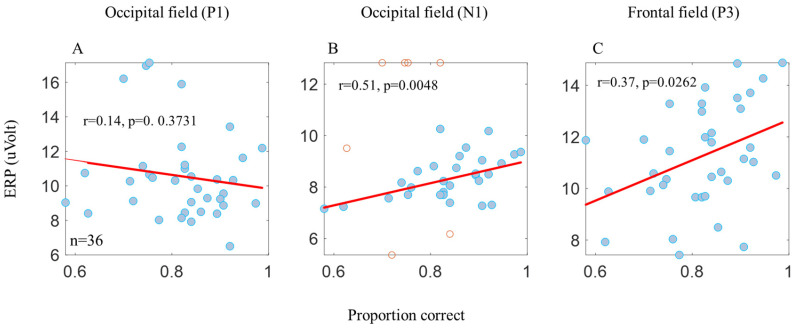
Pearson’s correlation between behavior and the ERP components. No significant correlation was found between the proportion correct and the P1 amplitude (**A**). A significant correlation was found between the proportion correct, the N1 amplitude (**B**), and the P3 amplitude (**C**). The N1 and P1 components of the occipital electrodes (the PO3, POZ, PO4, O1, OZ, and O2 electrodes) and the P3 component of the frontal electrodes (the F1, FZ, and F2 electrodes). The blue dots in the graph represent the participant’s average data (*n* = 9) from the 4 conditions (*n* = 36). The correlation only applies to the blue dots. The red dots represent the outlier data according to Pearson’s correlation formula.

**Figure 4:**
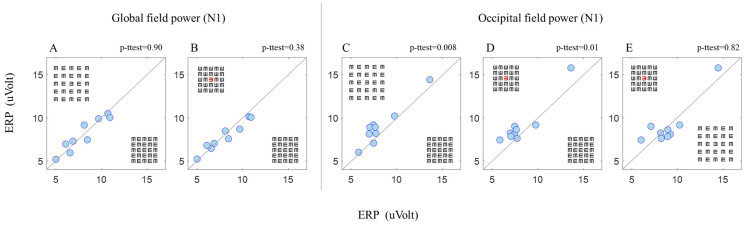
Global (refers to the average of all electrodes (64 electrodes)) field power (N1) between BC and BUC (**A**) and between BC and RC (**B**). Occipital field power (N1) between BC and BUC (**C**), between BC and RC (**D**), and between BUC and RC (**E**). The dots in the graph represent the participant’s average data (*n* = 9). Each dot in the graphs represents the average of the runs of each participant under two conditions indicated in each graph and separated by a diagonal line. The *p* value represents a statistical paired *t*-test.

EEGs were recorded from 64 passive tin electrodes (the Quik-Cap uses gel for application) arranged in standard locations across the scalp. (We performed our analyses on the main electrodes relevant to our study: PO3, POZ, PO4, O1, OZ, O2, F1, FZ, and F2, i.e., all analyses of P1 and N1 refer to the average of the occipital area electrodes (PO3, POZ, PO4, O1, OZ, and O2), and all analyses of P3 refer to the average of the frontal area electrodes (F1, FZ, and F2)). Since early EEG amplitudes, such as the P1 component, are sensitive to luminance and the stimulus size [34,50,51], we examined this component in the occipital region, represented by occipital (‘O’) electrodes. Also, since the P3 component is affected by attention and decision making, we examined this component in the frontal region, represented by frontal (‘F’) electrodes. The ERP results (Figure 2, the upper graphs) were obtained according to two main electrodes; OZ was considered as processing in the occipital area, and FZ was considered as processing in the frontal area. The EEGs were continuously sampled at 1 KHz and amplified with a SynAmps2/RT amplifier (Neuroscan, Victoria, Australia) with a bandpass of DC to 100 Hz. The data were low pass filtered at 30 Hz. For every condition, the average ERPs were computed over a 650 ms period per trial, with 150 trials per participant and 1350 trials per condition (one participant was not considered due to a technical fault in the recording). For each trial, the mean of 100 ms before the onset of the stimulus was taken as the baseline for the trial. The waveforms of the evoked responses (Figure 2), the correlations (Figure 3), and the global field power (Figure 4) were analyzed separately for each participant. Peak amplitudes were measured for a positive component, labeled as P1 (70–190 ms) and P3 (250–800 ms), and for a negative component, it was labeled as N1 (100–240 ms). Note that we found it appropriate to present only the graph of the correct answers because incorrect answers can result from a lack of attention, blinking, and so forth, which may add noise to the graph. Also, this method is compatible with the RT only presenting the results of the correct answers.

### 2.4. Data Analysis

After verifying that all of the data (proportion correct and log (RT)) were normally distributed and that the variances were homogenous (by Levene’s test), we used the two-factor ANOVA with replication test to compare the main groups (density and color). Also, we used a *t*-test, namely the Paired Two Sample for Means tool, to compare two specific parameters; the effect size (ES) is presented using Cohen’s d method. This method is used for comparing conditions. All statistical results were computed after a multiple comparison test was completed.

The calculated sample size in this study was based on the ‘Sample Size Justification’ [52] article and was calculated using G*Power 3.1.9.4 software, i.e., the sample size was calculated (post hoc) using the mean and SD (standard deviation) parameters (comparison between BC and RC was 1.57 for the proportion correct, 1.52 for the reaction time, and 1.89 for the N1 amplitude). All values showed that the required sample size was up to 10 participants. In addition, this sample size is consistent with previous studies from our lab [Sterkin et al., 2012 [35] (*n* = 8), Siman-Tov et al., 2021 [31] (*n* = 10)].

All correlation graphs refer to Pearson’s correlation, where each dot in the graphs represents the average runs of one participant.

Extreme value artifacts were rejected by denoting them as ‘bad blocks’: time regions around amplitudes exceeding a ±150 μVolt threshold were measured at the Mean Global Field Potential (MGFP) and +60 μVolts at the frontal channels (F7, Fz, and F8); samples at a time range of −200 to 800 ms were excluded. The following are the numbers of trials that were rejected for each condition in the occipital electrodes: BUC (191), BC (180), RUC (212), and RC (210) (i.e., around 15% of all of the trials). The following are the numbers of trials that were rejected in the frontal electrodes: BUC (161), BC (150), RUC (223), and RC (158) (i.e., around 13% of all of the trials).

## 3. Results

### 3.1. Psychophysics

**Figure 5:**
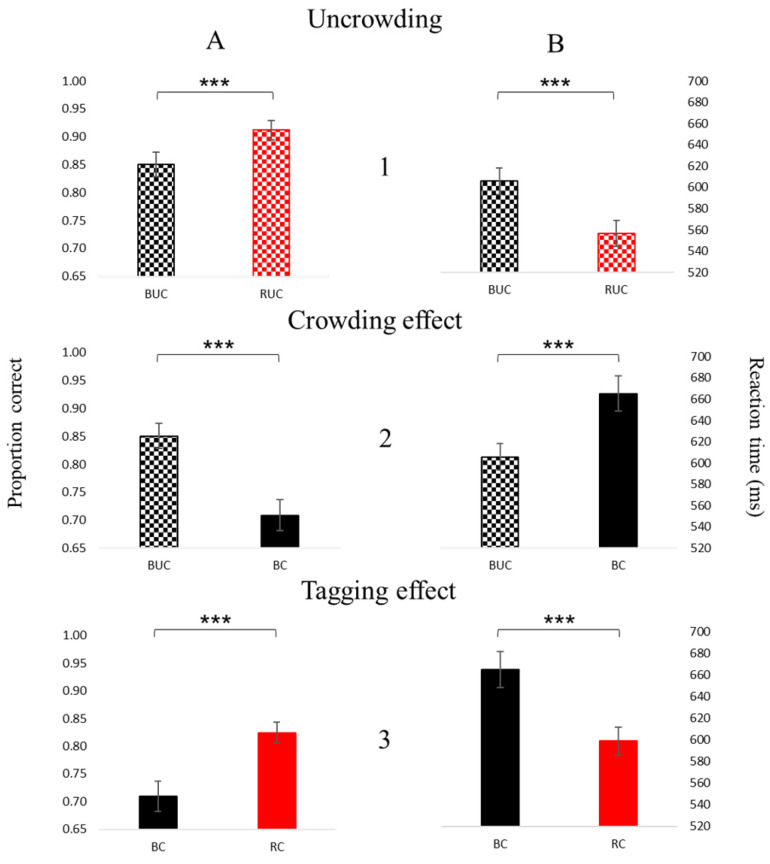
Psychophysics results: the proportion correct (A) and the reaction time (B) for the uncrowded (1), crowded (2), and tagging (3) effects. Comparisons between two conditions for each of the graphs (the proportion correct and the reaction time) are significant. The bars represent the mean across participants with SE (standard error) bars. Three asterisks represent *p* < 0.001.

The results (Figure 5) show that foveal crowding relative to an uncrowded condition reduces sensitivity (Figure 5(A2); t(9) = 7.73, *p* < 0.001, ES = 1.95) and increases the reaction time (RT) (Figure 5(B2); t(9) = 5.59, *p* < 0.001, ES = 1.41), whereas tagging reduces the crowding effect, i.e., improves sensitivity (Figure 5(A3); t(9) = 4.80, *p* < 0.001, ES = 1.62), and shortens the RT (Figure 5(B3); t(9) = 5.49, *p* < 0.001, ES = 1.56). In addition, the tagging improves sensitivity (Figure 5(A1); t(9) = 5.13, *p* < 0.001, ES = 1.04) and shortens the RT (Figure 5(B1); t(9) = 6.36, *p* < 0.001, ES = 1.39) even under uncrowding conditions. Table 1 presents an ANOVA test performed on the two main groups characterizing the stimuli, density, and color. We found a significantly different result under density (dense vs. spacious) and color (black vs. red) for the proportion correct and the reaction time, with no significant interaction between them (density and color). The ability to indicate the E direction was significantly reduced, and the reaction time was significantly increased under crowded (dense) compared to uncrowded (spacious) conditions. In addition, tagging (red) conditions improved the ability and the reaction time compared to black E. The fact that no interaction was found between density and color indicates that neither are required to reduce crowding.

**Figure 6:**
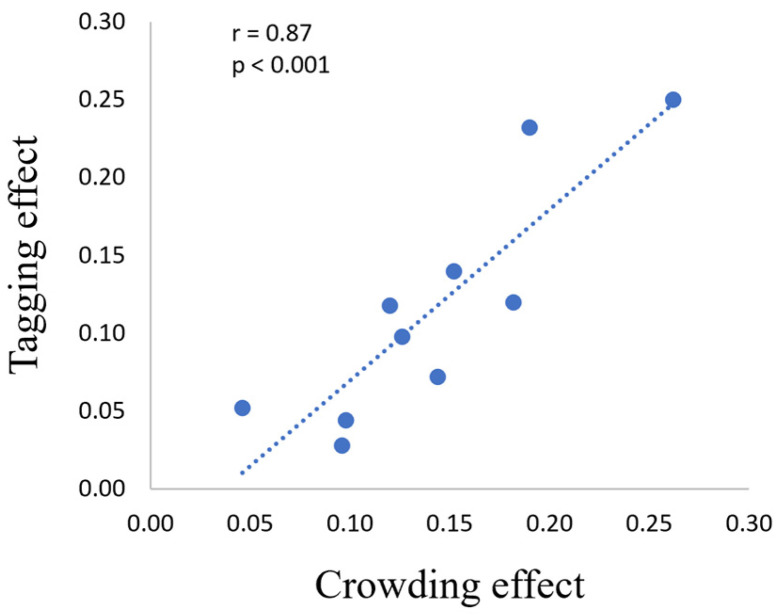
A high Pearson’s correlation for the psychophysical results: the crowding (reduction) [it represents the difference between BUC and BC conditions] and the tagging (improvement) [it represents the difference between RC and BC conditions] effects. The axis values are shown in proportion to the correct units. Each dot in the graph represents the difference in the average runs of each participant.

To understand whether a relationship exists between crowding and the tagging mechanism, we correlated the percentage of crowding reduction with the percentage of tagging improvement (Figure 6). Importantly, we found a significant correlation between them (r = 0.87, *p* < 0.001).

### 3.2. ERP

Note that the total energy levels of all stimuli were equal (according to luminance and contrast), but the matrix size differed; it was larger for the 1-letter spacing (uncrowded) and smaller for the 0.4-letter spacing (crowded). Table 2 presents an ERP ANOVA test performed on the two main groups characterizing their stimuli, density, and color. The ERP results (Figure 2) of P1 followed the physical properties, revealing a significant difference (Table 2A) between the smaller matrix (0.4-letter spacing, the black crowded (BC), the red crowded (RC)) versus the larger (1-letter spacing, BUC, RUC) matrix. However, no significant difference was observed due to tagging within the same matrix density (Table 2A). Also note that the P1 results, at a time window of 50–200 ms, do not correlate with the psychophysics results (Figure 3A). Thus, P1 does not follow the psychophysical results, which is consistent with the assumption that P1 represents the basic stimulus properties [34]. Therefore, apparently, the difference between the two matrix densities (dense and spacious) is due to the visual retinal density of the matrix [50], i.e., there is a higher amplitude for a dense matrix (see the Section 2).

However, we found a significant correlation between the N1 amplitude, measured at the occipital electrodes at a time window of 150–250 ms, and the psychophysical results (Figure 3B). This result is consistent with the assumption that N1 represents lateral interactions [35] and crowding [34]. 

It was interesting to discover that there is a significant correlation between the P3 amplitude, measured at the frontal electrodes at a time window of 250–650 ms, and the psychophysical results (Figure 3C).

A significant N1 amplitude difference was observed between BC and BUC (Figure 4C) and between BC and RC (Figure 4D). Note that this significant result for N1 was found in the occipital area only (Figure 4A,B showing no difference between BC and BUC and between BC and RC in the global area electrodes). However, no significant difference was observed between BUC and RC (Figure 4E). The above results of the N1 receive further statistical support in Table 2.

## 4. Discussion

Our previous studies reinforced our results, indicating that crowding also occurs in the fovea [31], and the previous results are consistent with the current results; foveal crowding (under a brief presentation time) relative to a single letter (uncrowded) reduces the sensitivity and increases the reaction time (RT), whereas tagging reduces the crowding effect, improves sensitivity, and shortens the RT (as well as improves binocular summation). We found a significant difference between the uncrowded (one letter spacing) and the tagging conditions (Figure 5(A1)), which slightly differs from our previous study [31]. The difference stems from the fact that, in the previous article, we presented a single letter without any distractors, whereas in this study, the uncrowded condition consisted of a matrix with one-letter spacing (see the Methods Section).

However, it is important to note that this difference between BUC (black uncrowded) and RUC (red uncrowded) is significant (t(9) = 5.13, *p* < 0.001, ES = 1.04, Figure 5(A1)) and that it is still smaller compared with the differences obtained under tagging conditions (t(9) = 4.80, *p* < 0.001, ES = 1.62 Figure 5(A3)). These results are consistent with the viewpoint that crowding is affected by the grouping effect; therefore, tagging breaks up the grouping and reduces the crowding effect [11,53,54].

The issues of the underlying mechanisms of crowding, the locus, and the relationship between crowding and other visual phenomena, such as pop-out and masking, have been widely studied [2]. The assumption that the crowding effect is processed early in the occipital area [9,17,18] was demonstrated by the result that the threshold elevation aftereffect was substantially reduced during crowding [55] and because crowding occurs when the target is presented to one eye and the flankers are presented to the other eye [56]. However, other studies assumed that crowding results from an attention deficit [20,21,22,23] based on the finding that crowding, like other attentional effects, is stronger in the upper visual field than in the lower visual field [20] and the fact that the polarity effect in crowding has a temporal resolution similar to that for attention [57]. Thus, these studies apparently assume that crowding occurs beyond V1. An ERP foveal crowding study that analyzed all electrodes (i.e., for all brain regions) [34] found that the suppression of the N1 component occurred under crowded conditions. The authors concluded that crowding occurs during higher-level visual processing.

Here, we chose to use EEG, aiming to better understand the mechanism underlying crowding and tagging and to determine whether they are related. We also examined the ERP results (Figure 2, the upper graphs) according to two main electrodes: OZ was considered as processing in the occipital area, and FZ was considered as processing in the frontal area [58]. The other analyses, ANOVA test (Table 2) correlations (Figure 3), and field power tests (Figure 4) were examined according to the occipital electrodes (PO3, POZ, PO4, O1, OZ, and O2) and the frontal electrodes (F1, FZ, and F2).

In the occipital area, we found that the P1 component represents the basic physical stimulus properties, i.e., the amplitude value density of the matrix is, respectively, is high for dense amplitudes and low for spacious amplitudes (see the Methods Section, stimuli, and procedure) (Figure 2). In addition, we did not find a significant correlation between the P1 amplitude and the proportion correct (Figure 3A). These P1 results are also consistent with those of Peng et al.’s 2018 study [30]. Note that the N1 characteristics can be affected by the P1 characteristics; however, despite the similarity between the P1 amplitudes under dense matrix conditions (BC and RC) and under spacious matrix conditions (BUC and RUC), we found that N1 displays different characteristics between these conditions (Figure 2A upper, comparing the two continuous black and red lines).I In addition, there is a significant and correlative difference for the N1 component between the stimuli according to the psychophysical results (Figure 3B). These results are consistent with the findings of Polat and colleagues in 2012 [35] and with those of Herzog and colleagues in 2014 [34], i.e., that N1 represents facilitatory lateral interactions and crowding. Therefore, in crowded conditions, when facilitation and identification are reduced, the N1 amplitude is also reduced. We also did not find a significant difference between BUC and RC (Figure 4E). This finding corresponds to and reinforces what we found in our previous study [31]. We hypothesized that, because our stimulus was presented in the central fovea, the first effect of tagging results in the matrix grouping being broken up by a different color (red). This hypothesis is consistent with the conclusions of Peng et al.’s study [30]. In addition, differences were found in the N1 value only under different conditions in the occipital electrode (Figure 4), which supports the hypothesis that crowding occurs at a lower visual processing level.

The second effect Is raising the level of attention, even though the stimulus is presented at the location where the participant is looking all the time (it corresponds to the common assumption that attention exists in the gaze area). This result is consistent with the conclusions of Poletti et al. [59]. We found a relationship not only between N1 and crowding (Figure 4C), but also between N1 and tagging (Figure 4D), i.e., the N1 amplitude is higher under uncrowded and tagging conditions compared with crowded conditions. To test this relationship, we examined the frontal area on the P3 component, which represents attention [38,39,40,41] and decision making [44] (at a time window of 250–650 ms) and therefore represents tagging. We found a high correlation between P3 and the proportion correct (Figure 3C), i.e., the P3 amplitude increases as the crowding effect decreases. These findings show that there is a mutual relationship between crowding and tagging. Thus, our ERP data may be consistent with the hypothesis that the awareness of crowding effect (or uncrowded) consists of representations distributed in different cortical locations [47].

## 5. Conclusions

Our study strengthens the assumption that N1 is reduced under crowded conditions; in addition, we examined N1 in the occipital area and for all electrodes and found that this reduction is only processed in the occipital region. This method strengthens the assumption that crowding probably occurs at a lower visual processing level. We also found a high correlation between P3 and the proportion correct, which led us to assume that there is also a connection between crowding and tagging. The finding that the P3 amplitude is reduced under crowding conditions and increases under tagging conditions strengthens the assumption that crowding serves as a bottleneck for awareness.

## Figures and Tables

**Table 1:** Statistics of psychophysics ANOVA: two factors with replication. The proportion correct (A) and the reaction time (Log) (B) for all four conditions.

A				
Proportion correct				
*Source of Variation*	*df*	*F*	*p-val* *ue*	*Effect Size*
Sample (dense vs. spacious)	1	30.0193	0.0000	0.45 (large)
Columns (black vs. red)	1	17.8622	0.0002	0.33 (large)
Interaction (density and color)	1	1.6663	0.2050	
Within	36			
Total	39			
**B**				
Reaction time (Log)				
*Source of Variation*	*df*	*F*	*p-val* *ue*	*Effect Size*
Sample (dense vs. spacious)	1	15.1317	0.0004	0.3 (large)
Columns (black vs. red)	1	19.6670	0.0001	0.35 (large)
Interaction (density and color)	1	0.2171	0.6441	
Within	36			
Total	39			

**Table 2:** Statistics of ERP ANOVA: two factors with replication. The P1 (occipital) (A), the N1 (occipital) (B), and the N1 (global field) (C) for all four conditions.

A				
Pl (Occipital)				
*Source of Variation*	*df*	*F*	*p-val* *ue*	*Effect Size*
Sample (dense vs. spacious)	1	6.2978	0.0173	0.16 (large)
Columns (black vs. red)	1	0.0514	0.8220	
Interaction (density and color)	1	0.4730	0.4965	
Within	32			
Total	35			
**B**				
Nl (Occipital)				
*Source of Variation*	*df*	*F*	*p-val* *ue*	*Effect Size*
Sample (dense vs. spacious)	1	10.8759	0.0024	0.25 (large)
Columns (black vs. red)	1	14.4843	0.0006	0.31 (large)
Interaction (density and color)	1	2.1420	0.1531	
Within	32			
Total	35			
**C**				
Nl (Global field)				
*Source of Variation*	*df*	*F*	*p-val* *ue*	
Sample (dense vs. spacious)	1	0.8967	0.3508	
Columns (black vs. red)	1	0.6066	0.4418	
Interaction (density and color)	1	0.5173	0.4772	
Within	32			
Total	35			

## Data Availability

The datasets used and/or analyzed during the current study are available from the corresponding author. The data are not publicly available due to the special encryption of the raw EEG data.
